# An association of cognitive impairment with diabetes and retinopathy in end stage renal disease patients under peritoneal dialysis

**DOI:** 10.1371/journal.pone.0183965

**Published:** 2017-08-31

**Authors:** Jin-Lan Liao, Zu-Ying Xiong, Zhi-Kai Yang, Li Hao, Gui-Ling Liu, Ye-Ping Ren, Qin Wang, Li-Ping Duan, Zhao-Xia Zheng, Jie Dong

**Affiliations:** 1 Renal Division, Peking University Shenzhen Hospital, Shenzhen, China; 2 Renal Division, Department of Medicine, Peking University First Hospital; Institute of Nephrology, Peking University; Key Laboratory of Renal Disease, Ministry of Health; Key Laboratory of Renal Disease, Ministry of Education, Beijing, China; 3 Renal Division, the Second Hospital of Anhui Medical University, Anhui, China; 4 Renal Division, the Second Affiliated Hospital of Harbin Medical University, Heilongjiang, China; 5 Renal Division, Handan Central Hospital, Hebei, China; Hospital Universitario de la Princesa, SPAIN

## Abstract

**Background:**

Diabetes and retinopathy have been considered as risk factors of cognitive impairment (CI) in previous studies. We investigated both of these two factors and their relationship with global and specific cognitive functions in end stage renal disease patients under peritoneal dialysis (PD).

**Methods:**

In this multicenter cross-sectional study, 424 clinically stable patients were enrolled from 5 PD units, who performed PD for at least three months and completed fundoscopy examination if they had diabetes. Global cognitive function was measured using the Modified Mini-Mental State Examination (3MS), Trail-Making Test forms A and B for executive function, and subtests of the Battery for the Assessment of Neuropsychological Status for immediate and delayed memory, visuospatial skills, and language ability.

**Results:**

PD Patients with DM and Retinopathy had significantly higher prevalence of CI, executive dysfunction, impaired immediate memory and visuospatial skill, compared with patients in non-DM group. By multivariate logistic regression analyses, DM and retinopathy rather than DM only were significantly associated with increased risk for CI, executive dysfunction, impaired immediate memory and visuospatial skill, odds ratios(ORs) and 95% confidence intervals were 2.09[1.11,3.92], 2.89[1.55,5.37], 2.16 [1.15,4.06] and 2.37[1.32,4.22], respectively (all P < 0.05).

**Conclusions:**

Diabetic PD patients with retinopathy were at two times risk for overall cognitive impairment, executive dysfunction, impaired immediate memory and visuospatial skill as compared to non-diabetic PD patients.

## Introduction

Peritoneal dialysis (PD) involves complex activities such as self-monitoring, self-care, lifestyle changes and adaptation to dialysis and medication regimens. All above are partly dependent on normal cognitive function [1] [2, 3]. Unfortunately, the prevalence of cognitive impairment is high: 27%–67% among patients with end stage renal disease (ESRD) [1, 4–6]. As cognitive impairment (CI) is an independent predictor of mortality[4] and technical survival [7]in dialysis patients, exploring the risk factors for CI is crucial in this situation.

Diabetes mellitus (DM) is known to be prevalent in chronic kidney disease, i.e.44% of patients with ESRD as reported by USRDS [8] and 37.3% of Chinese PD patients from our multi-center cohort study [9]. Of note, previous studies have indicated a link of diabetes to an increased risk of CI [10–12]. Diabetic participants with retinopathy perform worse on cognitive tests since retinopathy reflects micro-vascular abnormalities, possibly sharing similar metabolic and inflammatory pathways with CI [13–15].

This is an affiliated study of a multi-center cross-sectional survey on cognitive function in ESRD under PD. Relevant studies were published elsewhere [3, 16–18]. Although our previous data have shown that DM is weakly associated with general and specific CI [3, 17], further analysis on the additional impact of retinopathy on CI for diabetic PD patients has not been performed. To our knowledge, few data has been shown on this association in dialysis population. Therefore, we aimed to explore the relationship between DM, retinopathy and cognitive function in patients on PD.

## Patients and methods

### Patient selection and study design

Data used were pooled from five PD centers (Beijing, Heilongjiang, Hebei, Anhui, and Guangdong) located at 4 geographical regions (north, northwest, east, and south) in China, detailed study design was published at main research[18]. Database from each center were collected within strictly controlled Framework and were further inspected to ensure the integrity and accuracy. All investigators and staff members completed a training program which taught them the study methods and processes. A manual of detailed instructions for data collection was distributed. Also, the study was approved by the ethics committee of Peking University First Hospital. All participating patients signed written agreement for acknowledging their information to be stored on the hospital database and used in research. The investigator had no access information that could identify individual participants during or after data collection.

This study enrolled prevalent PD patients between March 2013 and March 2014. Inclusion criteria for participants were: age ≥18 years; had been undergoing PD ≥3 months and clinically stable; able to undergo all measurements and questionnaires as required. Patients were excluded if they had a systemic infection, acute cardiovascular events, active hepatitis, or cancer; had undergone surgery or experienced trauma in the month before the study; had any study-obstructive conditions (severe eyesight loss, language incompatibility illiteracy, preexisting dementia or confusion, various mental disorders, or upper limb disability); or no referral to ophthalmology and fundoscopy examination. All the participants received conventional glucose-based lactate-buffered PD solutions (Ultra bag, Baxter Healthcare, Guangzhou, China).

### Clinical characteristics

Demographics and comorbid conditions were recorded, including age, sex, education level, PD vintage, body mass index (BMI), systolic and diastolic blood pressure, primary kidney disease, the presence of diabetes mellitus (DM), history of cardiovascular disease (CVD) and Charlson index[19]. Mean arterial pressure was calculated. The degree of education was recorded as the highest school level at which a diploma was received (i.e., elementary school or lower, middle school, high school, or above high school). CVD was recorded in at least one of the following conditions: angina, congestive heart failure (New York Heart Association class III-IV), transient ischemic attack, history of myocardial infarction or cerebrovascular accident, and peripheral arterial disease[20]. Cerebrovascular accident was recorded separately.

### Laboratory methods

After overnight fasting while continuing PD therapy, participants had their venous blood sampled for routine and biochemical measurements. Biochemical data (including serum sodium, serum albumin, calcium, phosphate, triglycerides, total cholesterol, high-sensitivity C-reactive protein (hs-CRP), serum hemoglobin A_1_C (HbA_1_C) and hemoglobin) were gathered with an automatic Hitachi chemistry analyzer, and the mean value of measurements taken over the preceding 3 months was calculated. Residual kidney function was defined as the mean of residual creatinine and urea clearance from a 24-hour urine collection. Dialysis adequacy was defined as total Kt/V and creatinine clearance.

### Cognitive function and retinopathy

Overall cognitive function was measured by the modified Mini-Mental State Examination (3MS)[21]. We used a 3MS cutoff point of less than 75 for individuals with less than a high school education and a 3MS cutoff point of less than 80 for individuals with a high school education because the 3MS mean scores vary by education[22]. Specific cognitive functions measured were executive function (by the Trail-Making Test, forms A [Trails A] and B [Trails B]), immediate memory, delayed memory, visuospatial skill, and language ability by subtests of the Repeatable Battery for the Assessment of Neuropsychological Status (RBANS). Executive dysfunction was de fined as Trails A score more than 75 seconds and Trails B score more than 180 seconds.[23–25] In addition, subtests of RBANS were adopted to assess immediate memory (list learning and story memory), delayed memory (list recall, list recognition, story recall, and figure recall), visuospatial skill (figure copy), and language ability (picture naming and semantic fluency).[26]The reliability and validity of RBANS have already been established in Shanghai and Beijing populations [27, 28]. Raw scores were transferred to age-standardized T scores for all subtests of RBANS. T scores less than 1 standard deviation below the published mean in an education-grouped Chinese population were identified as impair for each test.[29] Neurocognitive battery was assessed after patients had a meal just in case of hypoglycemia.

Participants were referred to the ophthalmologist, and examined with fundoscopy after mydriasis if they had diabetes. We collected their final diagnosis on retinopathy and registered it to the Charlson comorbidity index. Definition of retinopathy is an ocular manifestation of systemic disease as seen in diabetes or hypertension [30]. The retinal changes included classic retinal vascular changes in diabetes and hypertension (i.e., diabetic and hypertensive retinopathy), isolated retinopathy signs in individuals with diabetes or hypertension (e.g., microaneurysm, retinal hemorrhage, or cotton wool spot).

### Statistical analysis

Continuous data were presented as mean ± standard deviation except for PD vintage, residual kidney function, and hs-CRP level, which were presented as median with interquartile range due to high skew. Categorical variables were presented as proportions. Patients were divided into 3 groups: non-diabetes mellitus (non-DM), DM with or without retinopathy. One-way analysis of variance, or *x*^2^ was used to compare differences in demographic and biochemistry data and parameters of general and specific cognitive function between groups.

The effects of DM with or without retinopathy on overall and specific CI were examined respectively by using multivariable logistic regression analysis adjusting for recognized confounders. Three models of logistic regression analysis models were built to examine whether DM with or without retinopathy could correlate to the risk of cognitive impairment, executive dysfunction, impaired immediate and delayed memory, visuospatial skill, and language ability, respectively. Model1 included demographic data (age, sex, education level, BMI, DM with retinopathy). Model2 involved Model1 and laboratory parameters (serum albumin, sodium, total cholesterol, hs-CRP, and residual kidney function). Model 3 contained Model1 and Model2 plus cerebrovascular disease. All probabilities were two-tailed, and the level of significance was set at 0.05. Odds ratios (ORs) and 95% confidence intervals were calculated. Statistical analysis was performed using SPSS for Windows software, version 20.0 (SPSS Inc.).

## Results

### Basic characteristics

**Fig 1:**
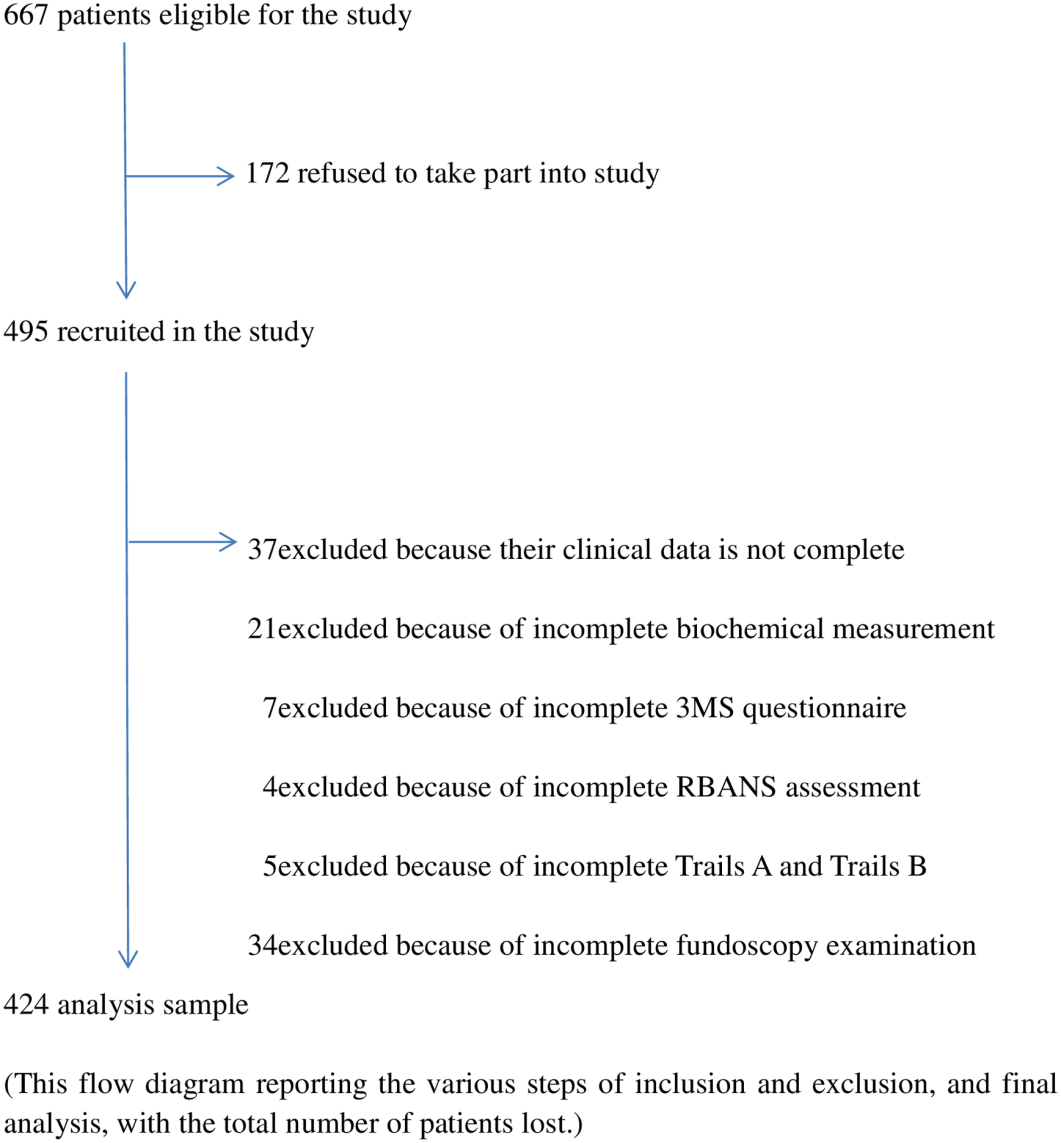
Selection and exclusion process of participants.

Among 667 patients screened for enrollment, 495(74.2%) gave consent and 71(14.3%) did not meet eligibility criteria, leaving 424(85.7%) patients who had cognitive testing and performed fundus examination (Fig 1). Demographic and laboratory results for our participants were slightly different with our previous study because the sample size [3, 17, 18]. The basic data was in accordance with general characteristics of the PD population in China [27]. For instance, these enrolled patients had a mean age of 52.7±14.3 years, PD vintages of 33.2±27.5 months, BMI of 22.9±3.4 kg/m^2^, serum albumin of 36.0±5.5 g/L, and serum hemoglobin A1C (HbA1C) of 6.3±1.0%. Among these patients, 72.4% were non-diabetic, 8.5% were diabetes only, and 19.1% were diabetes with retinopathy, 21.9% had a history of CVD, 9.3% had a history of cerebrovascular accident (Table 1).

**Table 1:** Clinical characteristics according to diabetes and retinopathy.

Variables	Total	non DM	DM	DM and Retinopathy	P
**No. of patients**	424 (100%)	307 (72.4%)	36(8.5%)	81 (19.1%)	—
**Age, y**	52.7 y4.3	50.1 y5.1^c^	61.0 y.1	58.8 y.3^e^	<0.001
**Male sex**	217(51.2%)	147(47.9%)^b^	26(72.2%)	44(54.3%)	0.02
**PD vintage, months**	33.2intag	34.0intag	27.0intag	32.1intag	0.31
**Cardiovascular disease**	93 (21.9%)	53(17.3%)^a^	12(33.3%)	28(34.6%)^d^	0.001
**Cerebrovascular accident**	38(9.3%)	22(7.5%)	2(6.1%)	14(17.3%)^d^	0.02
**Level of education**					0.2
**≤Elementary school**	84(19.8%)	64(20.8%)	4(11.1%)	16(19.8%)	
**Middle school**	124(29.2%)	88(28.7%)	11(30.6%)	25(30.9%)	
**High School**	123(29.0%)	80(26.1%)	15(41.7%)	28(34.6%)	
**>High school**	93(21.9%)	75(24.4%)	6(16.7%)	12(14.8%)	
**BMI, kg/m^2^**	22.9±3.4	22.5±3.4^a^	23.9±3.4	24.1±3.4^e^	<0.001
**MAP, mmHg**	99.9 mmHg	101.3mmHg0	96.73mmH	96.33mmHg^d^	<0.001
**Hemoglobin, g/L**	104.2lobin	102.3lobin	108.2lobin	109.8lobin^d^	<0.001
**HbA1C, %**	6.31C,	6.01C, ^a^	6.41C,	6.61C, %^d^	<0.01
**Serum albumin, g/L**	36.0m al	35.7m5.9	36.9m5.9	36.5m5.9	0.27
**Triglyceride, mmol/L**	2.0glyc	1.8glyc	2.1glyc	2.0glyc	0.2
**Total cholesterol, mmol/L**	4.8al c	4.7al c	4.9al c	4.9al c	0.57
**Serum sodium, mmol/L**	139.0 sod	139.3 sod	138.2 sod	138.6 sod	0.05
**Calcium, mmol/L**	2.3cium	2.3cium	2.2cium	2.3cium	0.07
**Phosphate, mmol/L**	1.7spha	1.7spha	1.7spha	1.7spha	0.99
**hs-CRP,mg/L**	3.2[1.1,8.7]	2.9 [0.9,8.4]	2.6[1.3,8.1]	4.0[1.7,12.1]	0.67
**Residual kidney function, ml/min/per 1.73 m^2^**	2.0[0.0,6.0]	2.0[0.0,5.9]	2.5[0.0,7.7]	1.1[0.0,5.2]	0.1
**Total Kt/V**	1.9al K	1.9al K	1.8al K	1.8al K	0.01
**Total CLcr, L/wk/1.73 m^2^**	58.2l CLc	58.7l CLc	59.1l CLc	56.1l CLc	0.48

Values for categorical variables are given as number (percentage); values for continuous variables, as mean± standard deviation or median. [Interquartile range]. Abbreviations: BMI, body mass index; CLcr, creatinine clearance per week; DM, diabetes mellitus; hs-CRP, high-sensitivity C-reactive protein; Kt/V, urea clearance per week; MAP, mean arterial pressure; PD, peritoneal dialysis

^a^ P<0.05,

^b^ P <0.01,

^c^ P<0.001, non DM group vs. DM group;

^d^ P<0.01,

^e^ P<0.001, non DM group vs. DM and Retinopathy group

### Clinical characteristics according to DM and retinopathy

As shown in Table 1, diabetic patients with or without retinopathy were prone to be older, male, having higher proportion of cardiovascular disease and cerebrovascular accident, and higher BMI, hemoglobin, triglycerides level, and lower MAP levels as compared to non-diabetic patients. There were no significant differences in demographic and biochemistry data between DM only and DM with retinopathy groups (all P<0.05). Although the Kt/V values differed between non-DM and DM group, dialysis adequacy was clinically acceptable for both.

### DM, retinopathy and cognitive function

Among all 424 subjects, the prevalence of CI was 27.4%. As compared to non-DM group, patients with diabetes and retinopathy had significantly lower scores for 3MS, immediate memory and visuospatial function, and longer completion time on executive tests. Accordingly, they had significantly higher prevalence of cognitive impairment, executive dysfunction, impaired immediate memory and visuospatial skill than non-DM patients (Table 2). However, diabetic patients did not perform worse in overall and specific cognitive function than non-diabetic patient. The scores for delayed memory and language ability were not significantly different between groups (Table 2).

**Table 2:** Cognitive function parameters according to diabetes and retinopathy disease.

Variables	Total	non DM	DM	DM and Retinopathy	P
**N (%)**	424	307(72.4%)	36 (8.5%)	81(19.1%)	—
**3MS score**	83.4score	84.3score	85.5scor^f^	79.1scor1^d^	0.004
**Cognitive impairment**	113(27.4%)	72(24.2%)	7(21.2%)^g^	34(42%)^d^	0.004
**Trails A,s**	88.1ls A,	82.0ls A,	92.6ls A,	109.9s A,s^d^	0.008
**Trails B,s**	207.4s B,s1	191.8s B,s3	204.9s B,s	268.0s B,s1^e^	0.001
**Executive dysfunction**	127(32%)	73(25.2%)	13(41.9%)	41(53.9%)^e^	<0.001
**Immediate memory score**	73.1diate	74.9diate	70.0diate	67.7diate^d^	0.003
**Impaired immediate memory**	275(67.2%)	185(62.7%)	26(76.5%)	64(80.0%)^d^	0.007
**Delayed memory score**	88.6yed m	88.9yed m	89.4yed m	87.0yed m	0.66
**Impaired delayed memory**	83(21.3%)	62(21.8%)	7(22.6%)	14(18.9%)	0.85
**Language ability score**	92.4uage	93.2uage	92.4uage	89.2uage	0.08
**Impaired language ability**	69(16.8%)	43(14.5%)	8(23.5%)	18(22.5%)	0.13
**Visuospatial skill score**	85.2ospat	87.8ospat	84.3ospat	75.6ospat^e^	<0.001
**Impaired visuospatial skill**	194(48.9%)	129(44.3%)	16(51.6%)	49(65.3%)^d^	0.005

^d^ P<0.01,

^e^ P<0.001, non DM group vs. DM and Retinopathy group;

^f^ P<0.05,

^g^ P<0.01, DM group vs. DM and Retinopathy group

**Fig 2:**
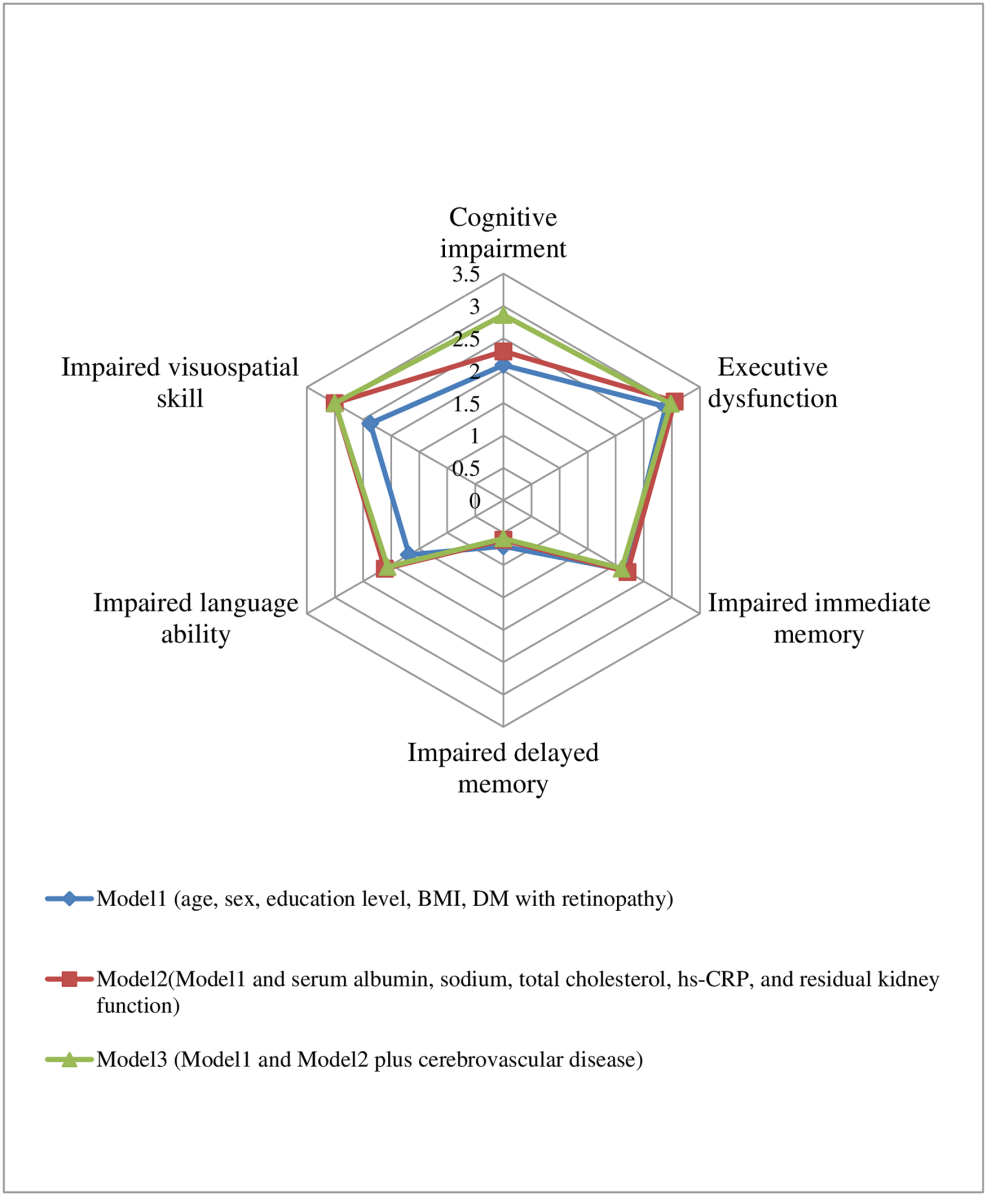
Comparision odd ratio values of DM and retinopathy in three models.

We further determined whether DM with or without retinopathy was associated with an increased risk of cognitive decline in PD patients (Table 3). In Model 1, DM with retinopathy was significantly associated with increased risk for CI, executive dysfunction, impaired immediate memory and visuospatial skill, the odds ratios(ORs) and 95% confidence intervals were 2.09[1.11,3.92], 2.89[1.55,5.37], 2.16 [1.15,4.06] and 2.37[1.32,4.22], respectively (all P < 0.05). After adjusting for demographic and additionally clinical parameters or comorbid data, this result did not weaken, the OR values of DM and retinopathy which to be indicated executive dysfunction and impaired immediately memory was approximately (Fig 2). DM only did not show any influence on the global or specific cognitive impairment.

**Table 3:** The effect of diabetes and retinopathy on impaired cognitive function by multivariable logistic analysis.

Variables	Model 1		Model 2		Model 3	
	OR		OR		OR	
	(95%confidence interval)	P	(95%confidence interval)	P	(95%confidence interval)	P
**Cognitive impairment**						
DM	0.84(0.31,2.31)	0.74	0.82(0.29,2.30)	0.709	0.85(0.30,2.39)	0.76
DM and Retinopathy	2.09(1.11,3.92)	0.022	2.19(1.13,4.26)	0.021	2.15(1.10,4.20)	0.03
**Executive dysfunction**						
DM	1.78(0.73,4.35)	0.206	1.71(0.68,4.31)	0.259	1.79(0.71,4.53)	0.22
DM and Retinopathy	2.89(1.55,5.37)	0.001	2.88(1.51,5.52)	0.001	2.82(1.46,5.42)	0.002
**Impaired immediate memory**						
DM	1.85(0.78,4.42)	0.164	1.74(0.71,4.22)	0.22	1.86(0.76,4.54)	0.17
DM and Retinopathy	2.16(1.15,4.06)	0.016	2.17(1.13,4.20)	0.02	2.05(1.05,3.98)	0.04
**Impaired delayed memory**						
DM	1.12(0.43,2.95)	0.81	1.03(0.40,2.79)	0.954	1.04(0.38,2.82)	0.94
DM and Retinopathy	0.71(0.35,1.44)	0.347	0.59(0.59,1.28)	0.169	0.57(0.27,1.21)	0.14
**Impaired language ability**						
DM	1.93(0.77,4.82)	0.159	1.91(0.72,5.09)	0.196	1.81(0.64,5.12)	0.19
DM and Retinopathy	1.68(0.87,3.23)	0.121	1.86(0.93,3.74)	0.081	1.81(0.90,3.65)	0.1
**Impaired visuospatial skill**						
DM	1.34(0.60,2.99)	0.474	1.50(0.64,3.50)	0.347	1.508(0.65,3.52)	0.34
DM and Retinopathy	2.37(1.32,4.22)	0.004	2.60(1.41,4.79)	0.002	2.58(1.40,4.77)	0.002

Model 1: age, sex, education level, BMI, DM and retinopathy; Model 2:, Model 1 plus serum albumin level, sodium level, total cholesterol level, high-sensitivity C-reactive protein level, and residual kidney function; Model3: Model 2 plus cerebrovascular disease; Abbreviations: OR: odd ratio.

## Discussion

The main findings of our current study showed that DM with retinopathy was associated with worse performance on cognitive function than non-DM group, including overall cognitive function, executive function, immediate memory and visuospatial skill rather than delayed memory and language ability. Diabetic patients without retinopathy performed similarly on overall and specific cognitive function as compared to non-diabetic patients although they were older, having higher BMI and HbA1C level.

As shown in prospectively longitudinal studies, there is an association of diabetes with the onset of dementia and CI as compared with those without diabetes [11, 12]. Yet in our study, PD patients with diabetes did not perform worse than non-diabetes on global and special cognitive function after multivariate adjustment. There are several possible reasons for the result. First, multiple risk factors in relation to ESRD, such as inflammation, endothelial dysfunction, atherosclerosis, vascular calcification and hyperhomocysteinemia [31–35], also play roles in the development and progression of CI. The accumulation of advanced glycosylation end products triggers vascular endothelial dysfunction, increases permeability of blood vessels and causes hardening [36–38]; by which the mechanisms correlate with CI in T2DM may react in ESRD patients under PD [39–41]. PD vintage of non-DM group was longer than DM group in our subject; this can be interpreted as the hypothesis that historical glucose exposure of non-DM group was not low. Under these circumstances, the influence of DM only on the risk for CI might be weak in this population. This phenomenon is also in accordance with our previous data indicating diabetic without inflammation had comparable survival rate as compared to non-diabetic PD patients[9]. Second, the proportion of diabetic PD patients without retinopathy just presented to be 8.5%, which was significantly lower than that in a previous study from a western PD population (41%)[1]. Therefore, we cannot preclude the possibility that sample size of our study is not large enough to reject the null hypothesis. Third, patients with acute comorbidities or any study-obstructive conditions were excluded from this study. It is possible that frail individuals with DM or the imminent dropout cases were not examined with cognitive function based on this cross-sectional dataset. To clarify this issue, the further longitudinal study with large sample size to explore the risk of DM on new onset CI should be performed in dialysis population.

The risk of CI significantly increased in patients with DM and retinopathy in this study. They were comparatively older and having higher BMI, higher proportion of cerebrovascular and cardiovascular comorbidities, poor glucose controlled as compared to non-diabetic patients. All above are risk factors of CI, which performed worse as DM, combined with retinopathy rather than DM only. In addition, recent studies have showed a significantly independent effect of retinopathy on CI. There was a near threefold increased risk of CI after coronary artery bypass grafting in patients with diabetic retinopathy compared to those without [42]. More recently, Chronic Renal Insufficiency Cohort (CRIC) Study also indicated that the odds of CI on executive function, attention, and naming were 2–3 times higher in CKD patients with retinopathy than those without [43]. This association reflects the possible homology between retinal and cerebral microvasculature and the potential role of vascular injury in cognitive decline. Ono and colleagues reported that retinal circulation may be an indicator of cerebrovascular circulation[44] since the retina develops from the forebrain, their share similar embryological origin, size, structure and physiological characteristics (including the blood-brain and blood-retinal barrier)[45, 46]. However, when we included cerebrovascular disease as confounding factors into the model, the effect of DM and retinopathy on the risk for CI was limited changed. This finding needs to be further explored to determine whether retinopathy has an independent effect, not related to cerebrovascular disease, on the occurrence of CI. Based on the data, it inspired us that use of screening cognitive tests may be considered as a relatively simple and non-invasive assessment to provide a unique ‘window’ to detect a potential decline in cognitive function for the dialysis patient.

The strengths of our study included not only an extensive battery of six cognitive tests to assess impairment on multiple cognitive domains, but also the first multicenter study to examine the relationship between DM, retinopathy and cognitive impairment among PD patients. Most recognized confounders were taken into account into multivariate regression models to analyze the independent association of DM, retinopathy and CI.

However, the study was not without limitations. First of all, we did not use standardized photographic evaluation of retinal images to ascertain and grading retinopathy lesions [46–50]. The association of retinopathy in varying degree and CI could not be determined. Secondly, we did not measure visual acuity, which was shown to be associated with poor cognitive test scores in older people [51]. Executive function (the Trails A and Trails B), and some items in 3MS require adequate sight to provide a valid measurement of cognitive function. Although we excluded the patients with severe eyesight loss, it remained a question whether the results were confounded by concurrent changes in lens opacity, visual acuity, or contrast sensitivity in individuals with retinopathy[52]. Thirdly, in spite of our study population was consistent with the previous series studies [3, 17, 18], but selection criteria were varied for the study purposes. It was inevitable that the slight differences of experimental parameters, which were due to the different sample size and grouping. Last but not the least, one important confounding factor in the relationship between DM, retinopathy and CI was cerebrovascular disease, which was drawn from the Charlson Index rather than an objective examination such as computer tomography or magnetic resonance imaging of brain. This would result in the underestimation for the prevalence of cerebrovascular disease. However, these limitations would not lead to a reject to a null hypothesis.

In summary, we observed that diabetic PD patients with retinopathy met with two times risk for overall cognitive impairment, executive dysfunction, impaired immediate memory and visuospatial skill than non-diabetic PD patients. By contrast, diabetic PD patients without retinopathy performed comparable general and specific cognitive function. Although we could not determine whether retinopathy is a pathogenic factor of CI due to the nature of this cross-sectional study, our initial findings indicated the association of cognitive impairment with diabetes and retinopathy in PD population. Given the possible prognostic importance of retinopathy in relation to cognitive dysfunction in PD patients, further research to elucidate causes of these signs is needed.

## Supporting information

S1 File Figure file quality report: 2017-07-19. (PDF)

S2 File *PLOS ONE* clinical studies checklist. (DOCX)

S3 File STROBE statement. (DOCX)
