# Supplementary material for: An association of cognitive impairment with diabetes and retinopathy in end stage renal disease patients under peritoneal dialysis
Source: PLoS One. 2017 Aug 31;12(8):e0183965. doi: 10.1371/journal.pone.0183965 (PMC5578503; doi:10.1371/journal.pone.0183965)
Supplement: S1 File — (PDF) [file pone.0183965.s001.pdf]

PLOS

Figure file quality report: 2017-07-19				
Original Filename	PACE Filename	Status	Error Detail(s)	PACE Adjustments
Fig2 (1)-1		✓	• No Error	• DOC file is converted to a TIF for submission. Please inspect the tif version for image clarity and content.
Fig1 (5)-1		✓	• No Error	• DOC file is converted to a TIF for submission. Please inspect the tif version for image clarity and content.
